# A High-Throughput Immune-Oncology Screen Identifies Immunostimulatory Properties of Cytotoxic Chemotherapy Agents in TNBC

**DOI:** 10.3390/cancers16234075

**Published:** 2024-12-05

**Authors:** Kennady K. Bullock, Thomas Hasaka, Emily Days, Joshua A. Bauer, Patricia A. Ward, Ann Richmond

**Affiliations:** 1 Department of Pharmacology, Vanderbilt University School of Medicine, Nashville, TN 37232, USA; kennady.k.bullock@vanderbilt.edu (K.K.B.); patricia.a.ward@vanderbilt.edu (P.A.W.); 2 Vanderbilt Institute of Chemical Biology, Vanderbilt University, Nashville, TN 37232, USA; thomas.p.hasaka@vanderbilt.edu (T.H.); emily.days@vanderbilt.edu (E.D.); joshua.a.bauer@vanderbilt.edu (J.A.B.); 3 High-Throughput Screening Facility, Vanderbilt University, Nashville, TN 37232, USA; 4 Department of Biochemistry, Vanderbilt University School of Medicine, Nashville, TN 37232, USA; 5 Department of Veterans Affairs, Tennessee Valley Healthcare System, Nashville, TN 37232, USA

**Keywords:** breast cancer, immune response, CD8+ T-cells, cytotoxic chemotherapy

## Abstract

**Background:** Triple-negative breast cancers (TNBCs) typically have a greater immune cell infiltrate and are more likely to respond to immune checkpoint inhibition (ICI) than ER+ or HER2+ breast cancers. However, there is a crucial need to optimize combining chemotherapy strategies with ICI to enhance overall survival in TNBC. **Methods:** Therefore, we developed a high-throughput co-culture screening assay to identify compounds that enhance CD8+ T-cell-mediated tumor cell cytotoxicity. Over 400 FDA-approved compounds or agents under investigation for oncology indications were included in the screening library. **Results:** Four chemotherapy agents were chosen as priority hits for mechanistic follow-up due to their ability to enhance T-cell-mediated cytotoxicity at multiple doses and multiple time points: paclitaxel, bleomycin sulfate, ispinesib, and etoposide. Lead compounds affected the expression of MHCI, MHCII, and PD-L1 and induced markers of immunogenic cell death (extracellular ATP or HMGB1). **Conclusions:** Based on the ability to increase tumor cell susceptibility to T-cell-mediated cytotoxicity while minimizing T-cell toxicity, bleomycin was identified as the most promising lead candidate. Overall, the results of these studies provide mechanistic insight into potential new chemotherapy partners to enhance anti-PD-1 efficacy in TNBC patients.

## Simple Summary

Many breast cancers do not respond to immune checkpoint inhibitor therapy, even when combined with the standard of care chemotherapy. Here, we sought to identify compounds that enhance T-cell-mediated toxicity in tumor cells using a high-throughput screen of known anti-cancer drugs. Beginning with a library of over 400 FDA-approved or investigational compounds, we chose four chemotherapy agents for detailed mechanistic follow-up. We describe potential immunostimulatory properties of paclitaxel, bleomycin sulfate, ispinisib, and etoposide. Ultimately, these studies provide insight into the potential immunostimulatory properties of the chosen follow-up compounds and suggest avenues for future investigations into optimizing chemotherapy partners to use with ICI.

## 1. Introduction

**Figure 1:**
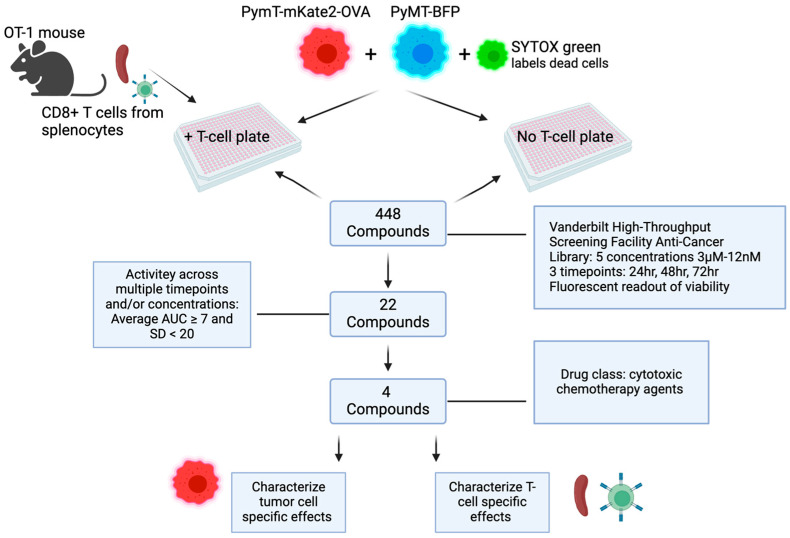
Schematic of experimental workflow from initial high-throughput screening strategy to validation of compounds of interest.

Triple-negative breast cancer (TNBC) is a heterogenous subtype accounting for about 10% of breast cancer (BC) cases in the United States annually [1]. TNBCs are characterized by a lack of HER2 amplification and a lack of expression of the hormone receptors for estrogen and progesterone (HR-). The immune checkpoint inhibitor anti-PD-1 was approved for clinical use in the adjuvant and neoadjuvant settings in combination with physician’s choice chemotherapy for TNBC based on results from the KEYNOTE-355 and KEYNOTE-522 trials, respectively. Although anti-PD-1 is now routinely used in TNBC treatment, there is great variability in responses between patients, and few achieve a durable response. For example, in the KEYNOTE-355 trial, the addition of anti-PD-1 improved progression-free survival (PFS) by about 4 months (9.7 vs. 5.6 months) [2]. The taxanes, nanoparticle albumin-bound paclitaxel (nab-PTX) or paclitaxel (PTX), are common chemotherapy partners used with anti-PD-1. PTX often requires pretreatment with steroids to counteract hypersensitivity reactions caused by the drug solvent—a polyethylated castor oil—while nab-PTX does not require steroid pretreatment [3]. While originally defined by their ability to disrupt microtubule assembly during cell division, taxanes are further being explored for their ability to induce immunogenic cell death (ICD) [4]. However, there is a concern that dose-dense chemotherapy regimens or the steroid premedication required for tolerance of PTX may have immunosuppressive effects in non-responding patients. For example, TNBC patients after taxane neoadjuvant chemotherapy exhibited decreased immune gene expression patterns [5]. Consequently, there is great interest in identifying optimal chemotherapy or targeted therapy approaches to use in combination with anti-PD-1. In this study, we utilized a novel, high-throughput immune-oncology screening platform to identify candidate compounds from a library of anti-cancer compounds that may enhance T-cell-mediated cytotoxicity (Figure 1). Such compounds may be effective in combination with anti-PD-1. Of 448 starting compounds, we identified 22 high-priority compounds for follow-up based on the ability to enhance T-cell-mediated cytotoxicity across multiple concentrations and/or time points. Based upon drug class and clinical relevance, we further specified four key lead compounds for mechanistic characterization of immunostimulatory properties, including effects on CD8+ T-cell proliferation and cytokine profile, tumor cell antigen presentation, and induction of immunogenic cell death markers. These four compounds—paclitaxel (PTX), ispinesib (Isp), etoposide (Etop), and bleomycin sulfate (Bleo)—are traditional chemotherapies with well-characterized cytotoxic mechanisms. PTX is considered the standard of care in combination with anti-PD-1 in TNBC [6]. Isp is a kinesin spindle protein inhibitor that causes cell-cycle arrest by preventing formation of the microtubule spindle complex [7]. Overexpression of kinesin proteins is associated with PTX resistance in patients with basal-like breast cancers [8], and in a screen of patient-derived organoids (PDOs) established from treatment-naïve TNBC biopsies, compounds targeting kinesin spindle proteins were effective in PDOs found to be non-responsive to taxanes [9]. While Isp was abandoned clinically because of a lack of efficacy, it was associated with fewer side effects than traditional taxanes like PTX [7] and may still be useful in the setting of PTX resistance. Etop is an FDA-approved topoisomerase II inhibitor used in the treatment of several cancers, including bladder, stomach, lung, prostate, testicular, and some lymphomas [10,11]. Etop plus platinum-based chemotherapy is used in combination with ICI in small cell lung cancer [12]. Oral Etop is being investigated as an alternative chemotherapy for heavily pretreated metastatic breast cancer patients, and early studies show this is a safe and potentially effective strategy for this patient population [13]. Interest in Etop was renewed during the COVID-19 pandemic since the drug can be given orally and does not rely on patients traveling to an infusion center [14]. Bleo is a glycopeptide antibiotic approved for use in treating head and neck squamous cell carcinoma, Hodgkin’s lymphoma, and testicular carcinoma. Preclinical studies show that Bleo may enhance CD8+ T-cell activity through inducing ICD [15]. In the CT26 mouse model of colon cancer, Bleo significantly reduced tumor growth, but this effect was lost if the mice were also treated with either a depleting anti-CD8 antibody or a depleting anti-IFNy antibody [16]. Since PTX, Isp, Etop, and Bleo are clinically used or tested chemotherapy agents, repurposing them to take advantage of newly identified immunostimulatory properties may be a promising direction for further investigation for TNBC treatment. In this work we describe dose-dependent immunostimulatory mechanisms of these compounds.

## 2. Materials and Methods

Cell lines and treatments. All cell lines were maintained in a humidified 5% CO_2_ incubator at 37 °C and tested for *mycoplasma* contamination regularly (e-Myco TM Plus, LiliF Diagnostics, Quezon City, Philippines). *Mycoplasma*-negative cells were used for all experiments. The PyMT breast cancer cells derived from an MMTV-PyMT breast tumor in a C57Bl/6 mouse were provided by the Hal Moses laboratory. Early passage PyMT-OVA and E0771-OVA cells were obtained from the laboratory of Jin Chen (Vanderbilt University, Nashville, TN, USA). The PyMT-mKate2-OVA cell line was generated through a lentiviral transfection of the PyMT-OVA cell line with mKate2, a far-red fluorescent reporter, and the PyMT-BFP cell line was generated through a lentiviral transfection of the PyMT cell line with a blue fluorescent reporter (BFP). The mKate2 and BFP lentiviral vectors were provided by Josh Bauer (Vanderbilt High-Throughput Screening Facility). mKate2-positive cells were selected based on resistance to blasticidin (10 μg/mL, InvivoGen, San Diego, CA, USA, Cat. #ant-bl-05), and BFP-positive cells were selected based upon hygromycin B resistance (500 μg/mL, RPI, Mt. Prospect, IL, USA, Cat. #H75020). The following compounds were used in cell treatment studies: paclitaxel (Selleckchem, Houston, TX, USA, Cat. #S1150), bleomycin sulfate (Selleckchem, Houston, TX, USA, Cat. #S1214), ispinisib (Selleckchem, Houston, TX, USA, Cat. #S1452), and etoposide (Selleckchem, Cat. #S1225).

OT-1 splenic T-cell isolation and culture. Spleens were harvested from 8- to 12-week-old OT-1 mice (Jax, Bar Harbor, ME, USA, strain #003831). OT-1 mice were housed and cared for in the AAALAC-accredited Vanderbilt University Medical Center Animal Research Facility and routinely monitored by laboratory and veterinary staff. Animals were euthanized by CO_2_ asphyxiation using isoflurane overdose followed by cervical dislocation in accordance with the *Guide for the Care and Use of Laboratory Animals.* Splenocytes were dissociated into a single-cell suspension using gentleMACS^TM^Dissociator (Miltenyi Biotec, Auburn, CA, USA, Cat. #130-093-235) and passed through a 70 μM filter. Red blood cell lysis was performed using sterile ACK lysing buffer (KD Medical, Columbia, MD, USA, Cat. #RGC-3015). CD8+ T-cells were isolated from the single-cell suspension via negative selection using the MagCellect^TM^ mouse CD8+ T cell isolation kit (R&D, Minneapolis, MN, USA, Cat. #MAGM203) or the EasySep^TM^ mouse CD8+ T cell isolation kit (Stem Cell Tech, Vancouver, BC, Canada, Cat. #19853). Successful enrichment of the CD8+ population was confirmed via flow cytometry (AF700, BioLegend, San Diego, CA, USA, Cat. #100729). OT-1 CD8+ T-cells were cultured in RPMI containing 10% FBS, 1% Pen Strep, IL-2 (10 ng/mL, BioLegend, San Diego, CA, USA,), and 2-BME (50 μM, Sigma, St. Louis, MO, USA, Cat. #M3148). OT-1 CD8+ T-cells were activated with OVA (257-264) peptide (5 μg/mL, Sigma, St. Louis, MO, USA Cat. #S7951) and/or Dynabeads^®^ Mouse T-activator CD3/CD28 (Gibco, Waltham, MA, USA, Cat. #11452D) according to the manufacturer’s instructions for 48 h prior to the addition to tumor cell cultures. The activation status of the CD8+ T-cells used in the HTS assay was confirmed by analyzing culture supernatants for IFNy by ELISA (Invitrogen, Waltham, MA, USA, Cat. #KMC4021).

HTS assay. A total of 500 PyMT-BFP and 500 PyMT-mKate2-OVA cells in 20 μL FluoroBrite^TM^ DMEM (Gibco, Waltham, MA, USA, Cat. #A1896701) were placed in each well in 384-well plates using the automated liquid handling multidrop Combi (Thermo Scientific, Waltham, MA, USA, Cat. #5840330). The next day, compounds from the 448 Anti-Cancer Compound Library (https://medschool.vanderbilt.edu/hts/compound-management/ (accessed on 8 June 2023) were resuspended in SYTOX green (Invitrogen, Cat. #S7020), then individually aliquoted to each well of tumor cells. The SYTOX green dye is taken up by dead cells. Compounds were tested across a range of 5 concentrations: 3 µM, 0.8 µM, 0.2 µM, 50 nM, and 12 nM. Immediately following the addition of compounds, either 500 prestimulated OT-1 CD8+ T-cells in 10 μL DMEM or 10 μL DMEM without T-cells was added per well. Whole-well images (4x were acquired at 24 h, 48 h, and 72 h treatment time points using the ImageXpress Micro XLS imaging system. Fluorescent imaging at each time point was used to assess cell viability in response to treatments in the presence or absence of T-cells. MetaXpress software (version 6.7) was used to analyze images and score live/dead PyMT-mKate2-OVA cells versus live/dead PyMT-BFP cells.

For each concentration of individual compounds, several ratios were calculated. The number of dead (STYOX+) PyMT-mKate2-OVA-expressing cells compared to dead (SYTOX+) PyMT-BFP cells was assessed per well. To account for antigen-specific T-cell effects compared to non-antigen-specific T-cell effects in the T-cell-containing plates, the following calculation was performed: $Kt=\frac{deadPyMTmKate2OVA}{deadPyMTBFP}.$ A Kt ratio > 1 represents greater antigen-specific, than non-antigen-specific T-cell cytotoxicity. To account for T-cell-independent, cytotoxic effects of the compounds, a similar ratio was calculated for each corresponding tumor-cell-only plate: $Kn=\frac{deadPyMTmKate2OVA}{deadPyMTBFP}$. Theoretically, we would expect this ratio to be 1, as the cytotoxicity of the compounds should be independent of whether or not the PyMT cells express the OVA antigen. A final ratio, Tf, comparing the T-cell plate to the tumor-cell-only plate was calculated: $Tf=\frac{Kt}{Kn}$. If Tf is greater than 1, meaning Kt > Kc, this suggests enhanced, antigen-specific T-cell-mediated cytotoxicity in that treatment condition. Tf was plotted versus concentration for each compound at each time point to calculate the area under the curve (AUC). The AUC value provides a composite measure of all Tf values for all concentrations tested for a particular time point. The average AUC and SD across time points were calculated, and compounds with an average AUC ≥ 7 were identified as high-priority for follow-up since they exhibited enhanced T-cell-mediated cytotoxicity across multiple concentrations and/or time points.

Viability assays. PyMT-OVA cells were seeded at a density of 0.01 × 10^6^ cells per well in 96-well flat-bottom plates (Genesee Scientific, El Cajon, CA, USA, Cat. #25-109MP) in DMEM (Gibco, Waltham, MA, USA, Cat. #12491015). The following day, media were aspirated, the cells were treated with the compound of interest at 2× desired concentration in 100 μL, and either 100 μL of T-cell media (RPMI 10%FBS, 1%PenStrep, IL-2 (10 ng/mL, BioLegend, SanDiego, CA, USA) and 2-BME (50 μM, Sigma, Cat. #M3148) or 100 μL of T-cells media with 0.002 × 10^6^, CD3/CD28 (Gibco, Cat. #11452D) preactivated CD8+ T-cells were added to each well. After 48 h of co-culture, the medium, including the T-cells in suspension, was aspirated from each well. Phenol-red-free DMEM (100 µL) and 100 µL of CellTiter-Glo^®^ luminescent cell viability reagent (Promega, Madison, WI, USA, Cat. #G7572) were added per well. CD8+ T-cell-only wells were included to specifically assess the compound toxicity on the T-cell population. Luminescence was read on a Promega GloMax Discover plate reader, and values were normalized to media-only and DMSO control wells to assess tumor cell viability after co-culture.

Flow cytometry. All flow data were collected using a 5-Laser Fortessa at the Vanderbilt Flow Cytometry Shared Resource. Data were analyzed using FLowJo 10.5.3 software. Apoptosis was assessed via staining for Annexin-V/FITC (Invitrogen, Cat. #11-8005-74), and cell death was assessed by 7-AAD (Invitrogen, Waltham, MA, USA, Cat. #00-6993-50) according to the manufacturer’s instructions using flow cytometry. Prior to the addition to co-culture, CD8+ T-cells were labeled with 25 μM CellTracker™ Blue CMF2HC (Invitrogen, Waltham, MA, USA, Cat. #C12881) according to the manufacturer’s instructions.

To assess CD8+ cell proliferation, OT-1 splenocytes were labeled with CellTrace Deep Red (Invitrogen, Waltham, MA, USA, Cat. # C34553), stimulated with Dynabeads^®^ Mouse T-activator CD3/CD28 (Gibco, Waltham, MA, USA, Cat. #11452D) and treated with compounds of interest at 10 nM or 50 nM concentrations. Wells were sampled at 24 h, 48 h, and 72 h time points, and cells were stained using the following antibodies: CD3/PerCpCy5.5 (Biolegend, San Diego, CA, USA, Cat. #100217, 1:200) and CD8/PE (Biolegend, Cat. #100707, 1:200). Live/Dead-Ghost Dye Violet 540 (Tonbo Biosciences, San Diego, CA, USA, Cat. #13-0870-T100) was used to exclude dead cells. Proliferation was assessed via measuring the intensity of APC fluorophore over time.

PyMT-OVA tumor cells were treated with compounds of interest and stained for flow cytometry with the following antibodies: MHCI(H2Kb)/APC (Biolegend, Cat. #116517, 1:100), MHCII/AF700 (Biolegend, San Diego, CA, USA, Cat. #107622, 1:100), and PDL1/PE (Biolegend, SanDiego, CA, USA, Cat. #124307, 1:100). Live/Dead-Ghost Dye Violet 540 (Tonbo Biosciences, San Diego, CA, USA, Cat. #13-0870-T100) was used to exclude dead cells.

Immunogenic cell death marker assays. PyMT-OVA tumor cells were seeded at a density of 0.01 × 10^6^ cells per well in 96-well flat-bottom plates in complete phenol-red-free DMEM. Extracellular ATP was assessed via luminescence using the RealTime-Glo Extracellular ATP assay according to manufacturer instructions (Promega, Madison, WI, USA, Cat. #GA5010). HMGB1 was measured using the Lumit high mobility group box 1 protein (HMGB1) Human/Mouse Immunoassay (Promega, Madison, WI, USA, Cat. #W6110) according to the manufacturer’s instructions.

3D culture assays. PyMT-OVA tumor cells were grown as spheroids in complete DMEM + 5% Matrigel (Corning, Corning, NY, USA, Cat. #356234) in 24-well or 96-well ultra-low attachment plates (Corning, Cat. #3473 and #3474). Images were acquired using an EVOS M7000 at 20× or 10× and analyzed using FIJI 3 software. For 24-well plates, cells were seeded at a density of 50,000 tumor cells per well, and for 96-well plates, cells were seeded at a density of 2500 tumor cells per well. Prior to imaging, tumor cells were stained with DAPI (300 nM, ThermoFisher, Waltham, MA, USA, Cat. #D1306). When indicated, the T-cells were stained with CellTracker Deep Red according to the manufacturer’s instructions (ThermoFisher, Waltham, MA, USA, Cat. #C34565).

## 3. Results

### 3.1. Compound Screen of 448 Drugs Across Three Time Points Identifies Four Lead Compounds

**Figure 2:**
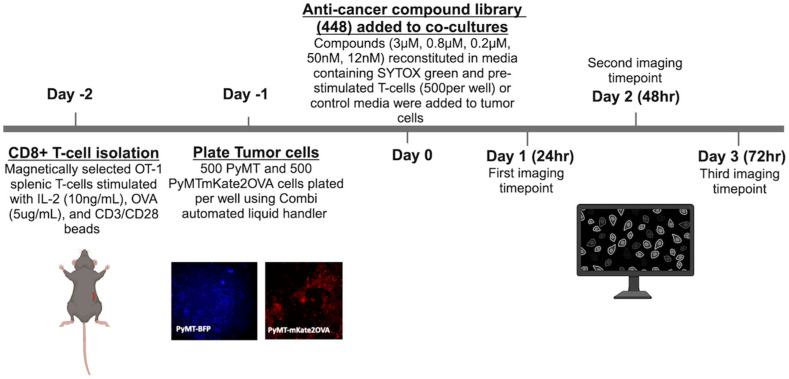
Schematic of high-throughput screening experimental timeline.

**Figure 3:**
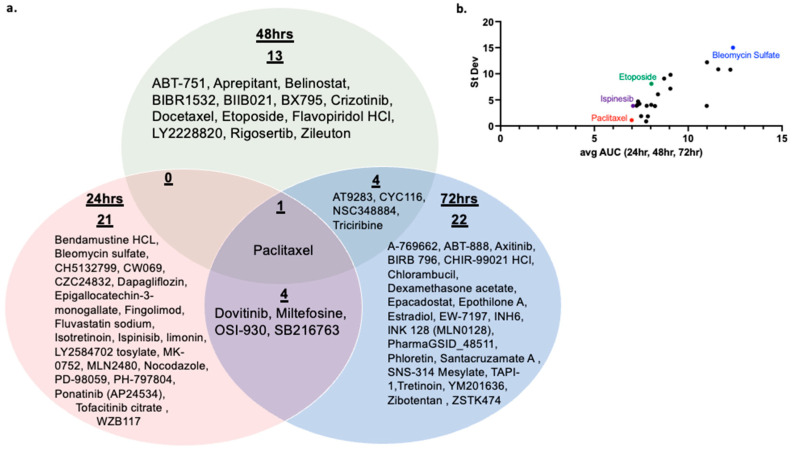
Selection of high-priority compounds (**a**) Compounds with a Tf > 3 at more than one concentration for each time point (**b**) Average AUC of Tf v concentration across three time points plotted v standard deviation. Colored compounds chosen for detailed mechanistic follow-up due to clinical relevance.

Using a library of anti-cancer compounds, we sought to identify compounds that enhance T-cell-mediated tumor cell cytotoxicity that may be useful in combination with anti-PD-1 therapy in TNBC models. Compounds were tested at 5 concentrations (3 µM, 0.8 µM, 0.2 µM, 50 nM, and 12 nM) across three time points (24 h, 48 h, 72 h), either in the presence or absence of preactivated murine OT-1 CD8+ T-cells isolated as described in the Methods section. Briefly, two days prior to the addition of drugs to tumor cells (day-2), CD8+ T-cells were isolated from OT-1 mouse splenocytes as described in Methods (Figure 2). Enrichment for CD8+ T-cells after magnetic selection was confirmed via flow cytometry (Figure S1). Supernatants from unstimulated control and preactivated T-cells were analyzed by ELISA for secretion of IFNy to confirm the activation status of T-cells added to the co-culture (Figure S1). One day prior to the addition of drugs to cultures, 500 PyMT-mKate2-OVA or 500 PyMT-BFP cells in 20 μL FluoroBrite^TM^ DMEM were plated into 384-well plates. Immediately following the addition of compounds, either 500 prestimulated OT-1 CD8+ T-cells in 10 μL DMEM or 10 μL DMEM without T-cells was added per well. Cultures were incubated for 24, 48, or 72 h, and dead cells were evaluated based on SYTOX green staining. A Tf greater than 1 represents enhanced death in the PyMT-mKate2-OVA population in the presence of T-cells compared to the PyMT-BFP population. Therefore, a Tf > 1 suggests enhanced, antigen-specific T-cell-mediated cytotoxicity. Tf was plotted against log[drug] for each compound to calculate an AUC value for each of the three time points (Figure S2). Compounds with a Tf > 3 at more than one test concentration per time point are shown in Figure 3a and are of particular interest because T-cell-mediated cytotoxicity is enhanced at more than one concentration tested. To further prioritize compounds, the average AUC and SD representing all three time points were calculated for each compound. Compounds with an SD > 20 were discarded for high variability. Compounds with an average AUC ≥ 7 were considered high-priority compounds, as these compounds have a Tf > 3 at more than one time point and/or concentration (Figure 3b, Table 1), indicating enhanced T-cell-mediated cytotoxicity across a range of treatment concentrations and time points. AUC and SD values for all compounds tested in the screen can be found in Table S1. PTX, Bleo, Isp, and Etop were chosen as priority compounds for mechanistic follow-up based upon drug class and clinical relevance. All four compounds are cytotoxic chemotherapy agents that have been well studied and tested clinically. Paclitaxel is routinely used in combination with anti-PD-1 for TNBC [6].

### 3.2. Confirmation of Increased T-Cell-Mediated Cytotoxicity

**Figure 4:**
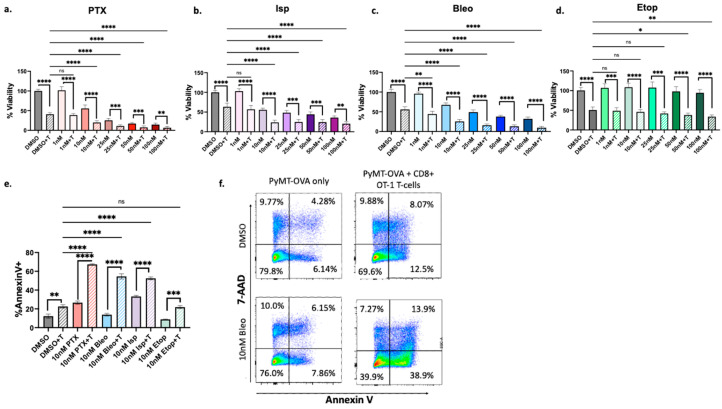
Confirmation of increased T-cell-mediated cytotoxicity. CellTiterGlo viability assay of PyMT-OVA cells after 48 h of treatment of (**a**) PTX (**b**) Isp (**c**) Bleo (**d**) Etop with or without CD8+ T-cells co-cultured at a 1:5 (T-cell:tumor cell) ratio. T-cell groups were compared to DMSO+T using a one-way ANOVA with Tukey’s post hoc analysis. For each treatment concentration, T-cell versus no T-cell conditions were compared using an unpaired *t*-test. ns: no significance, * = *p* < 0.05, ** = *p* < 0.01, *** = *p* < 0.001, **** = *p* < 0.0001 (**e**). Apoptosis assessed via FACS analysis of staining for Annexin V and 7-AAD. CD8+ T-cells stained with CellTrace blue and excluded by gating (**f**). representative flow plots of Annexin V and 7-AAD staining of PyMT-OVA tumor cells.

We next used two additional methods to confirm that the four priority compounds enhance T-cell-mediated cytotoxicity as detected in the initial high-throughput screen. CellTiter-Glo^®^ viability assays of PyMT-OVA cells cultured with or without OT-1 CD8+ T-cells were performed as the first validation method. In this assay, intracellular ATP detected after cells are washed and lysed is used as a measure of metabolically active, viable cells. A decrease in luminescent signal is interpreted as a decrease in intracellular ATP and a decrease in metabolic activity and cell viability. T-cells were removed prior to CellTiter-Glo^®^ analysis by removing and replacing the culture media at the experiment endpoint so as to only measure PyMT-OVA cell viability. In all conditions tested, samples treated with T-cells exhibited significantly reduced tumor cell viability compared to each corresponding treatment without T-cells. Samples treated with T-cells plus PTX (PTX+T) at 10 nM, 25 nM, 50 nM, or 100 nM exhibited significantly reduced tumor cell viability compared to samples treated with DMSO and T-cells (DMSO+T) (*p* < 0.0001) (Figure 4a). Similarly, samples treated with Isp+T exhibited significantly reduced tumor cell viability compared to DMSO+T at Isp concentrations of 10 nM, 25 nM, 50 nM, and 100 nM (*p* < 0.0001) (Figure 4b). Samples treated with Bleo+T exhibited significantly reduced tumor cell viability compared to DMSO+T at all of the concentrations tested (1 nM *p* < 0.01, 10 nM-100 nM *p* < 0.0001) (Figure 4c). Samples treated with Etop+T also exhibited significantly reduced tumor cell viability only at Etop concentrations of 50 nM (*p* < 0.05) and 100 nM (*p* < 0.01) (Figure 4d).

Evidence of enhanced apoptosis in the presence of both T-cells and the compound of interest was used as a secondary validation approach. PyMT-OVA tumor cells were cultured with or without prestimulated, fluorescently labeled OT-1 splenic CD8+ T-cells and treated with the indicated compounds for 48 h. After 48 h, the co-cultured cells were collected, and apoptosis was assessed via flow cytometry as described in the Methods section by staining for Annexin V and 7-AAD. The CD8+ T-cells were labeled with a CellTrace dye and therefore could be excluded from analysis by gating, allowing analysis of apoptosis specifically in the tumor cell population. Significantly more tumor cells were positive for Annexin V, a marker of apoptosis, in the presence of compound plus T-cell compared to compound alone for PTX (*p* < 0.0001), Bleo (*p* < 0.0001), Isp (*p* < 0.0001), and Etop (*p* < 0.001), confirming the increased T-cell-mediated cytotoxicity observed in the high-throughput assay (Figure 4e,f). Importantly, significantly more tumor cells were positive for Annexin V in the PTX+T, Bleo+T, and Isp+T groups compared to DMSO+T (*p* < 0.0001) (Figure 4e,f). No significant differences were observed in 7-AAD positivity, indicating that the increases in apoptosis observed at 48 h are early, rather than late-stage apoptosis.

### 3.3. Dose-Dependent Effects of Lead Compounds on Antigen Presentation by Tumor Cells

**Figure 5:**
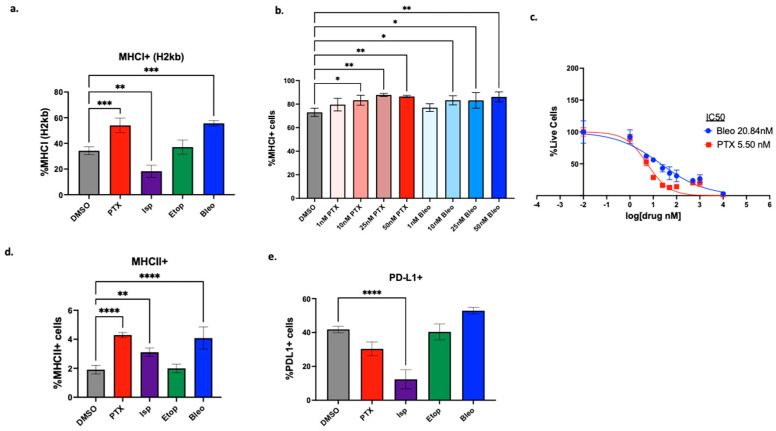
Effect of lead compounds on MHCI, MHCII, and PD-L1 expression by tumor cells. (**a**) PyMT-OVA tumor cells were treated with indicated compounds at 50 nM for 48 h, and MHCI (H2kb) expression was assessed via flow cytometry. (**b**) PTX and Bleo tested at a range of doses for 48 h. MHCI induction assessed via flow cytometry. (**c**) CellTiterBlue viability assay of OT-1 CD8+ T-cells treated with indicated compounds for 48 h. IC50 values were calculated using a non-linear regression of the data plotted at log10[drug] versus fluorescence using Prism version 10.1.0. (**d**) MHCII expression assessed via flow cytometry after 48 h of treatment with indicated compounds at 50 nM. (**e**) PD-L1 expression assessed via flow cytometry after 48 h of treatment of indicated compounds at 50 nM. Treatment groups were compared to the DMSO control using a one-way ANOVA and Tukey’s post hoc analysis. * = *p* < 0.05, ** = *p* < 0.01, *** = *p* < 0.001, **** = *p* < 0.0001.

The increased T-cell-mediated cytotoxicity detected in the high-throughput and apoptosis assays may occur through two distinct mechanisms; compounds may be affecting tumor cells in such a way that they are more susceptible to T-cell killing, or they may be directly affecting T-cell function. To test the hypothesis that the compounds enhance tumor cell susceptibility to T-cell killing, we first probed for induction of antigen presentation. PyMT-OVA tumor cells were treated with DMSO or 50 nM of PTX, Isp, Bleo, or Etop for 48 h. Surface expression of MHCI (H2kb) was assessed via flow cytometry. PTX and Bleo significantly increased H2kb expression compared to the DMSO control (*p* < 0.001), while Isp significantly decreased H2kb expression (*p* < 0.01) (Figure 5a). Since enhanced tumor cell death was observed with the combination of T-cells plus compound at doses as low as 1 nM for Bleo and 10 nM for PTX (Figure 4a,c), the dose dependency of MHCI induction was further explored. Starting at 10 nM, Bleo and PTX significantly enhance MHCI expression compared to the DMSO control (Figure 5b). Importantly, 10 nM is below the IC50 of Bleo for PyMT-OVA cells (Bleo 20.84 nM) (Figure 5c), indicating that Bleo may be able to stimulate T-cell-mediated cytotoxicity at sub-cytotoxic concentrations. MHCII surface expression was also assessed via flow cytometry since MHCII+ tumor cells were previously found to predict response to ICI in a study that used mass cytometry to profile TNBC tumors before, during, and after treatment [36]. PTX, Isp, and Bleo significantly increased MHCII expression (Figure 4d) (*p* < 0.0001, *p* < 0.01, *p* < 0.0001). PD-L1 was also assessed by flow cytometry since, in the late disease setting, TNBC tumors with high PD-L1 expression are more likely to benefit from ICI, though it has been reported that most of the PD-L1 is produced by stromal cells and not the tumor cells [37]. By FACS analysis of cultured PyMT cells, we observed that approximately 40% of the cells express PD-L1 at baseline (Figure 5e). Isp significantly decreased PD-L1 expression in PyMT tumor cells, while PTX, Bleo, and Etop had no significant effect after 48 h of treatment (Figure 5e).

### 3.4. Lead Compounds Induce Markers of ICD

**Figure 6:**
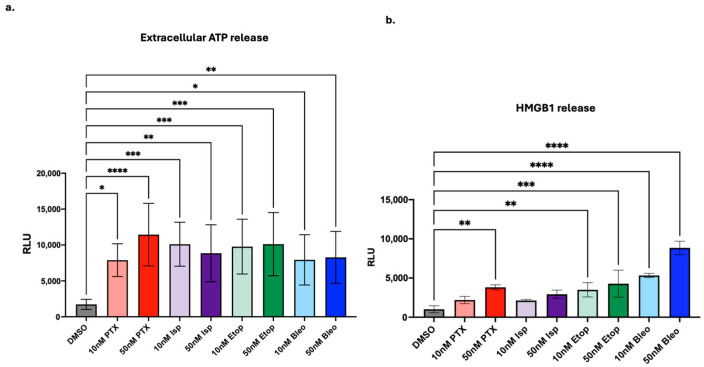
Induction of markers of ICD. (**a**) Extracellular ATP and (**b**) HMGB1 measured after 24 h of treatment with indicated compounds. Treatment conditions were compared to the DMSO control using a one-way ANOVA and Tukey’s post hoc analysis. * = *p* < 0.05, ** = *p* < 0.01, *** = *p* < 0.001, **** = *p* < 0.0001.

To probe how lead compounds may be affecting the immunogenicity of tumor cells, we next probed for markers of ICD. While ICD is a complex process best studied in in vivo models, we can probe for markers of ICD such as extracellular ATP and HMGB1, which may be detected in an in vitro setting [38]. Compared to DMSO-treated control, PTX, Isp, Etop, and Bleo significantly increase the release of extracellular ATP after 24 h of treatment at concentrations of 10 nM and 50 nM (Figure 6a). After 24 h of treatment, 50 nM, but not 10 nM, of PTX significantly increases HMGB1 release compared to the DMSO control (*p* < 0.01) (Figure 6b). Etop and Bleo both significantly increased HMGB1 release when used at 10 nM or 50 nM. Conversely, neither 10 nM nor 50 nM Isp significantly induces HMGB1 release, but it is possible that Isp would induce HMGB1 if tested at higher concentrations.

### 3.5. Dose-Dependent Effects of Lead Compounds on CD8+ T-Cell Activity

**Figure 7:**
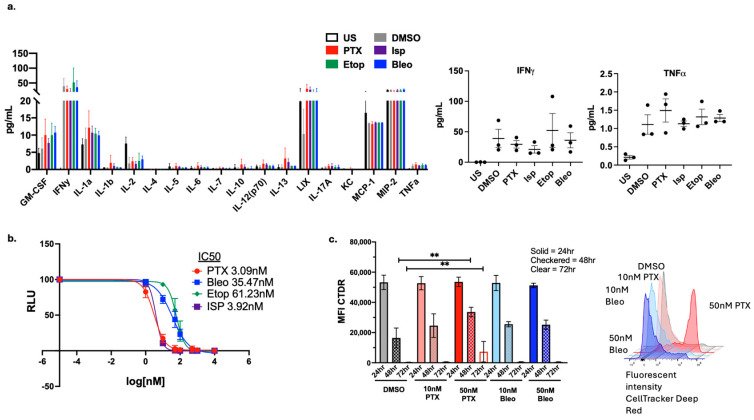
Effect of lead compounds on CD8+ T-cells. (**a**) Luminex cytokine array analysis of supernatants from OT-1 CD8+ T-cells treated with the indicated compounds at 50 nM for 48 h. n = 3 biological replicates. Data from IFNy and TNF are shown in expanded view. (**b**) CellTiterGlo viability assay of OT-1 CD8+ T-cells treated with indicated compounds for 48 h. IC50 values were calculated using a non-linear regression of the data plotted at log10[drug] versus luminescence using Prism version 10.1.0. Values presented as mean plus SD of three independent experiments. (**c**) CellTracker Assessment of OT-1 CD8+ T-cell proliferation via CellTracker proliferation assay. Mean fluorescent intensity (MFI) measured from samples at 24 h, 48 h, and 72 h. For each time point, treatment conditions were compared to the corresponding DMSO control using a one-way ANOVA and Tukey’s post hoc analysis. ** = *p* < 0.01. Representative plot of fluorescent intensity peak shown.

To investigate the second hypothesis that candidate compounds affect T-cell function, OT-1 CD8+ T-cells were treated with the indicated compounds for 48 h, and supernatants were collected for cytokine analysis. Supernatants were subject to Luminex cytokine analysis at the Vanderbilt Analytical Services Core using the high-sensitivity T-cell assay of 18 analytes. At the 48 h time point and 50 nM concentration of drug tested, the cytokine profile of T-cells was not significantly altered when compared to the DMSO control (Figure 7a). Of note, IFNγ and TNFα, two important cytokines involved in T-cell cytotoxicity, were not negatively impacted by drug treatment (Figure 7a). Moreover, the T-cells make substantial amounts of IL-1a in addition to cytokines that regulate myeloid cells (GM-CSF, LIX, MCP1, MCP2), though the levels of these cytokines were also not altered by drug treatment. To assess compound toxicity on CD8+ T-cells, the CellTiter-Glo^®^ luminescent cell viability assay was used to generate dose–response curves and calculate an IC50 for each compound after 48 h of treatment across a range of doses. PTX and Isp were more toxic to CD8+ T-cells than were Etop and Bleo, with IC50s of approximately 3 nM, 4 nM, 61 nM, and 35 nM, respectively (Figure 7b). The effects of PTX and Bleo on T-cell function were further assessed since they were the most effective at inducing tumor cell MHCI expression. CD8+ T-cell proliferation was assessed via flow cytometry using the CellTrace proliferation assay, where the intensity of the fluorescent dye decreases over time as cell divisions occur. Therefore, sustained fluorescent intensity indicates a reduction in cell proliferation. The 50 nM of Bleo did not significantly alter T-cell proliferation compared to the CD3/CD28-stimulated DMSO control at 48 or 72 h (Figure 7c). Conversely, 50 nM of PTX did significantly reduce T-cell proliferation at both 48 h and 72 h. To test whether the negative effects of PTX and Isp on T-cell proliferation were dose dependent, the proliferation assay was repeated using 10 nM of the indicated compounds. The lower dose of 10 nM did not significantly affect T-cell proliferation at either 48 h or 72 h (Figure 7c). Altogether, this indicates that the lead compounds do not enhance T-cell activation, but that at low doses of PTX and Bleo, CD8+ T-cell proliferation is not affected.

### 3.6. Validation in Additional Models

**Figure 8:**
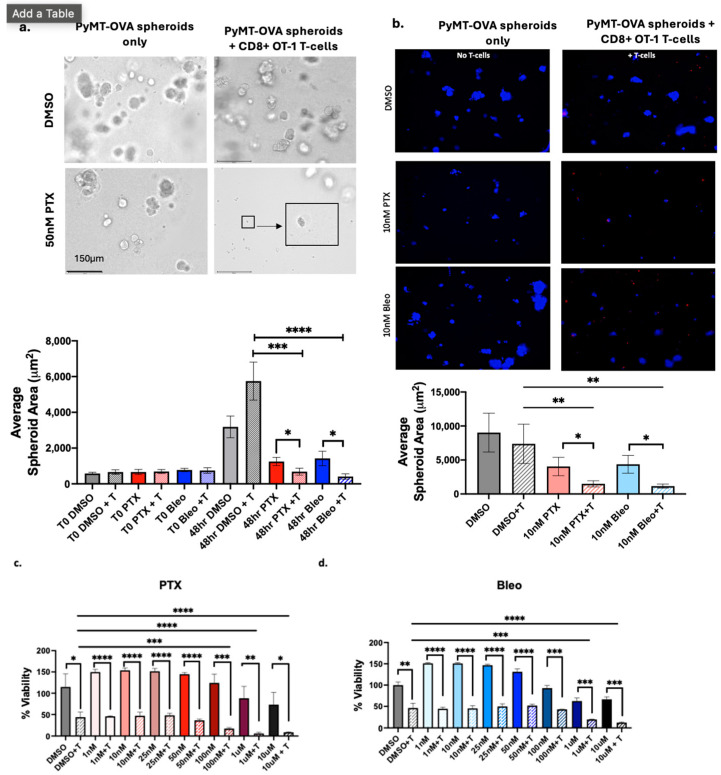
Validation of PTX and Bleo in 3D culture models. (**a**) PyMT-OVA cells grown as spheroids in 5% Matrigel in 24-well low-attachment plates and treated with indicated compounds for 48 h. Representative brightfield 20× images are shown. Scale bar = 150 µm. Enlarged insert showing small tumor spheroid. (**b**) PyMT-OVA cells grown as spheroids in 5% Matrigel in 96-well low-attachment plates. OT-1 CD8+ T-cells labeled with CellTracker Deep Red (red) and tumor cells labeled with DAPI (blue). Representative 10× fluorescent images are shown. Scale bar = 275 µm. For (**a**,**b**) PTX + T and Bleo + T compared to DMSO+T using a one-way ANOVA and Tukey’s post hoc analysis. For each treatment condition, T-cell versus no T-cell conditions were compared using an unpaired *t*-test. * = *p* < 0.05, ** = *p* < 0.01, *** = *p* < 0.001, **** = *p* < 0.0001. (**c**) CellTiterGlo viability assay of E0771-OVA cells after 48 h of treatment of PTX or (**d**) Bleo with or without CD8+ T-cells co-cultured at a 1:5 (T-cell:tumor cell) ratio. T-cell groups were compared to DMSO+T using a one-way ANOVA with Tukey’s post hoc analysis. For each treatment concentration, T-cell versus no T-cell condition was compared using an unpaired *t*-test. * = *p* < 0.05, ** = *p* < 0.01, *** = *p* < 0.001, **** = *p* < 0.0001.

We next sought to test the effectiveness of lead compounds in 3D culture models. As the current standard of care for use in combination with anti-PD-1 for TNBC, PTX was included in these studies. Bleo was chosen as the second compound for 3D validation since it increased expression of MHCI, MHCII, release of extracellular ATP, and release of HMGB1 at 10 nM. The amount of 10 nM is less than the IC50 of Bleo for both OT-1 CD8+ T-cells (Figure 7b, 35 nM) and PyMT-OVA tumor cells (Figure 5c, 20.84 nM). Therefore, Bleo is a promising candidate to be used at sub-cytotoxic concentrations to maximize immunostimulatory properties without negatively impacting T-cell function. PyMT-OVA cells were grown as spheroids in 5% Matrigel and cultured with or without OT-1 CD8+ T-cells in the presence of DMSO, 50 nM PTX, or 50 nM Bleo. Baseline images were acquired at time point zero prior to the addition of T-cells or drug treatment to confirm no significant differences in starting sizes of spheroids in each well. After 48 h, the average spheroid area of samples treated with PTX+T was significantly smaller than PTX alone (*p* < 0.05) and DMSO+T (*p* < 0.001) (Figure 8a). Similarly, the average spheroid area of samples treated with Bleo+T was significantly smaller than Bleo alone (*p* < 0.05) and DMSO+T (*p* < 0.0001) (Figure 8a). The experiment was repeated to test PTX and Bleo at 10 nM. PyMT-OVA spheroids were labeled with DAPI prior to imaging, and CD8+ OT-1 T-cells were labeled with CellTracker Deep Red, allowing us to confirm the presence of T-cells in the culture. The 10 nM PTX+T and 10 nM Bleo+T significantly reduced average spheroid size compared to DMSO+T (*p* < 0.01) and compared to the corresponding drug treatment without T (*p* < 0.05) (Figure 8b). Since TNBC is a heterogenous disease, we also sought to validate PTX and Bleo in the E0771-OVA cell line, an additional TNBC-like cell line. CellTiter-Glo^®^ viability assays of E0771-OVA cells cultured with or without OT-1 CD8+ T-cells were performed as described in the methods. In all conditions tested, samples treated with T-cells exhibited significantly reduced tumor cell viability compared to each corresponding treatment without T-cells (Figure 8c,d). Samples treated with PTX+T at 100 nM, 1 µM, and 10 µM exhibited significantly reduced tumor cell viability compared to samples treated with DMSO and T-cells (DMSO+T) (Figure 8c). Samples treated with Bleo+T at 1 µM and 10 µM exhibited significantly reduced tumor cell viability compared to each corresponding treatment without T-cells (Figure 8d).

## 4. Discussion

The study herein describes a high-throughput methodology to identify compounds that may enhance T-cell-mediated cytotoxicity and therefore are potential candidates for combination therapies with ICI. A total of 448 compounds from an anticancer compound library were tested across five concentrations and three time points for their ability to enhance T-cell-mediated cytotoxicity. The OT-1 model was used as a model of antigen-specific T-cell-mediated cytotoxicity. The OT-1 mouse is engineered to have transgenic T-cell receptors that specifically recognize the ovalbumin peptide (residues 257–264). While a useful tool, the OT-1 model is limited in its translational relevance because tumor antigen-specific T-cells may not be present in the TME. Nevertheless, the OT-1 model allows us to study T-cell to tumor cell interactions in isolation, and the findings presented in this work can be tested in non-antigen-specific models in future work. In the initial screen of 448 compounds, an average AUC was calculated across all three time points, and 22 compounds were identified as high-priority hits (Table 1, Figure 3b). Of these, PTX, Isp, Etop, and Bleo were chosen for detailed validation and follow-up analysis because they belong to a common drug class of cytotoxic chemotherapies. All four compounds exhibited enhancement of T-cell-mediated cytotoxicity in follow-up viability and apoptosis assays (Figure 4).

While four compounds were chosen for detailed follow-up analysis in the present work, the remaining 18 compounds may be the subject of future work (Table 1). Of particular interest are CYC116 and AT9283, which act as aurora kinase a/b inhibitors, and BIBR1532, a telomerase inhibitor. These act through a common mechanism of inducing cellular senescence. Therapy-induced senescence (TIS) was previously found to affect tumor-infiltrating lymphocytes and enhance the response to checkpoint inhibition in a mouse model of melanoma [39]. Rigosertib, a RAS mimetic, was also identified in this screen and may be the subject of further studies. It did not meet selection criteria to be included in the list of 22 high-priority compounds, but it did show evidence of enhancing T-cell-mediated cytotoxicity at the 48 h time point specifically (Figure 3a). In a mouse model of melanoma, rigosertib was previously found to induce ICD and synergize with ICI [40].

We first explored how the compounds of interest may affect the immunogenicity of the tumor cells. MHCI is a key molecule involved in presenting intracellular antigens to the cell surface, where they can be recognized by CD8+ T-cells as part of an adaptive immune response. MHCI downregulation is an immune evasive mechanism used by tumor cells. While NK cells can recognize cells missing this “self-signal” according to the missing-self hypothesis [41], MHCI downregulation is associated with resistance to ICI, and increasing MHCI expression is thought to improve ICI responses [42]. PTX and Bleo significantly increased MHCI expression at doses as low as 10 nM (Figure 5b). Bleo has previously been shown to increase MHCI expression in the B16-OVA mouse model of melanoma and to synergize with adoptive T-cell transfer therapy [43]. PTX, Isp, and Bleo also significantly increased expression of MHCII (Figure 5d). MHCII is primarily responsible for presenting antigens to CD4+ T-cells. In a study that characterized TNBC biopsies pre, during, and after treatment with anti-PD-1, pretreatment levels of MHCII+ tumor cells were a strong predictor of response [36]. An increase in MHCII expression does not explain the increased T-cell-mediated cytotoxicity observed in the HTS assay in the present study since CD4+ T-cells are not present in the co-culture assay. However, understanding how MHCII expression is affected will inform the suitability of combining these compounds with ICI in future studies and in vivo.

We next studied the ability of PTX, Bleo, Etop, and Isp to induce markers of ICD. Bleo and PTX have previously been found to induce markers of ICD such as HMGB1 [4], but here we test a variety of doses and show that 10 nM of Bleo or PTX is sufficient to induce ATP and HMGB1 release (Figure 6). Etop was also found to increase ATP and HMGB1 release at 10 nM and 50 nM (Figure 6). Etop was previously found to increase extracellular ATP and HMGB1 when used in combination with cisplatin, but not as a single agent, in a lung cancer cell line [44]. Isp has not previously been described as an ICD inducer, but in the present work, 10 nM and 50 nM significantly increased extracellular ATP release (Figure 6a). Beyond the proposed mechanisms of increasing T-cell-mediated cytotoxicity discussed so far, there are many other possible mechanistic explanations not explored in this work. For example, immunogenic modulation independent of induction of immunogenic cell death has been described for docetaxel [45]. This study found that docetaxel enhanced CD8+ T-cell-mediated tumor cell killing but did not induce traditional markers of ICD such as ATP and HMGB1 but rather relied primarily on calreticulin translocation to the membrane [45]. A similar mechanism may be at play here with PTX and Isp. Isp did not increase HMGB1 release, and PTX increased HMGB1 at 10 nM but not 50 nM (Figure 6), despite both compounds enhancing T-cell-mediated cytotoxicity at 10 nM and 50 nM (Figure 4a,b). While these studies are informative, ICD is best studied as an in vivo phenomenon, and future validation should include testing these compounds in mouse models of TNBC.

We next examined the effects of the compounds specifically on OT-1 CD8+ T-cells in vitro. No significant alterations in the cytokine profile of CD8+ T-cells were observed (Figure 7a); however, cytokine expression may need to be analyzed in the context of co-culture with tumor cells to observe composite alterations. The proliferation of CD8+ T-cells was also analyzed, and it was found that at 50 nM, Bleo does not negatively impact proliferation while PTX does decrease T cell proliferation. This problem can be mitigated by using PTX at the lower concentration of 10 nM, which is also effective (Figure 7c). Low-dose PTX delivered via nanoparticle formulation has previously been shown to increase proliferating T-cells in the E0771 murine model of TNBC [46]. A potential immunostimulatory role of PTX is further supported by single-cell analysis of TNBC patients, which showed that tumors that respond better to PTX exhibit an increase in CD8+ T cells, while non-responsive tumors exhibit increases in markers of immune cell exhaustion [47]. The CD8+ T-cell-specific toxicity of these compounds was further explored using CellTiter-Glo viability assays to calculate IC50s for each compound. While all four compounds are toxic to CD8+ T-cells at a high enough concentration (µM), Bleo and Etop are less toxic than PTX and Isp with IC50s of 35 nM, 61 nM, 3 nM, and 4 nM, respectively (Figure 7c).

Bleo and PTX were chosen for follow-up analysis in 3D culture models. PTX was included since it is the current standard of care for TNBC. Bleo was chosen as the most promising of the three remaining compounds since Bleo induced MHCI expression, MHCII expression, ATP release, and HMGB1 release at concentrations as low as 10 nM. Bleo also did not negatively impact CD8+ T-cell proliferation at 50 nM or 10 nM. Importantly, increased T-cell-mediated cytotoxicity was observed at concentrations less than the IC50 for both CD8+ T-cells (35 nM) and for PyMT-OVA tumor cells (20.84 nM). Bleo+T significantly reduced spheroid size compared to Bleo and DMSO+T when used at 50 nM and 10 nM (Figure 8). To date, Bleo has not been tested in combination with ICI in TNBC or any tumor type clinically. Toxicity remains a major concern that prevents the regular use of Bleo. Pulmonary toxicity involving pulmonary fibrosis is an adverse effect of Bleo reported to be experienced by about 10% of patients [48]. Lung and skin tissue lack expression of the hydrolase responsible for inactivating Bleo [35]. Respiratory immune-related adverse events, including interstitial lung disease and pneumonia, are also reported for ICIs [49], and there would be concern of compounding these risk factors. However, targeted delivery strategies or refinement of dosing schedules may mitigate the risk of toxicity. Preclinical studies loading Bleo into exosomes or tumor-targeting nanoparticles show promise in reducing toxicity and increasing drug delivery into the cytoplasm [50,51]. Furthermore, pingyangmycin, a glycopeptide antibiotic in the bleomycin family, has been shown to synergize with anti-PD-1 therapy in preclinical murine models of TNBC and melanoma without inducing pulmonary toxicities [52]. When Bleo and PTX were tested in an additional TNBC-like cell line, the E0771-OVA, both compounds enhanced T-cell-mediated cytotoxicity, albeit at much higher concentrations than what was required in the PyMT-OVA cell line studies. Whereas 10 nM of PTX or Bleo were sufficient to enhance T-cell-mediated cytotoxicity in the PyMT-OVA cell line (Figure 4a,c), 100 nM and 1 µM, respectively, were required to enhance T-cell-mediated cytotoxicity in the E0771-OVA cell line (Figure 8c,d). This is likely due to baseline differences in the two cell lines’ sensitivities to PTX and Bleo. The E0771-OVA are less sensitive to PTX and Bleo in vitro, and it has previously been shown that in in vivo mouse tumor models, E0771 tumors are less sensitive to PTX than are PyMT tumors [53]. This could be due to differences in drug efflux mechanisms in the different cell lines [54]. and further highlights the importance of validating findings and refining doses in multiple different TNBC models.

## 5. Conclusions

Overall, this work describes an HTS workflow designed to identify compounds that enhance T-cell-mediated cytotoxicity (Figure 1). The anticancer compound library from Vanderbilt University’s High Throughput Screening core was utilized in this study, but the workflow can be adapted to screen other libraries as well as other cell types. PTX, Bleo, Etop, and Isp were identified and validated as compounds that enhance T-cell-mediated cytotoxicity. Dose-dependent immunostimulatory effects were described, contributing to the growing body of literature working to identify optimal chemotherapy partners for use in combination with ICI. The PyMT-OVA cell line was tested here, but given the heterogeneity of TNBC, it will be useful to utilize additional TNBC models, including human TNBC organoids, in the future to further elucidate the potential of using these compounds to potentially enhance response to ICI in TNBC.

## Figures and Tables

**Table 1:** List of compounds with SD < 20 and AUC ≥ 7.

Compound	AUC	SD	Drug Class	Ref
Paclitaxel	6.99	1.11	Taxane chemotherapy	[17]
Ispinesib (Sb-715992)	7.06	3.84	Kinesin spindle protein inhibitor	[7]
MK-2866 (GTx-024)	7.25	3.88	Nonsteroidal selective androgen receptor modulator	[18]
GDC-0623	7.31	4.09	MEK1 inhibitor	[19]
Tretinoin (Aberela)	7.33	4.7	Vitamin A derivative	[20]
AT9283	7.40	4.24	Aurora kinase A/B and Jak2 inhibitor	[21]
BIBR1532	7.49	1.88	Telomerase inhibitor	[22]
Crizotinib (PF-02341066)	7.76	0.87	c-MET and ALK inhibitor	[23]
NSC348884	7.79	3.85	Nucleophosphmin inhibitor	[24]
YM201636	7.85	1.83	PIKfyve inhibitor	[25]
Miltefosine	8.03	4.04	Alkylphosphocholine	[26]
Etoposide (VP-16)	8.04	8.08	Topoisomerase II inhibitor	[11]
INH6	8.23	3.81	Nek2/Hec1 inhibitor	[27]
Divalproex sodium	8.39	6.07	Anti-epileptic	[28]
Isoretinoin	8.72	9.08	Vitamin A derivative	[20]
Phloretin	9.05	7.15	Natural dihydrochalcone	[29]
MK-0752	9.06	9.79	*γ*-secretase inhibitor	[30]
OSI-930	10.99	3.85	c-Kit inhibitor	[31]
Y-27632 2HCI	11.00	12.19	ROCK1/2 inhibitor	[32]
CYC116	11.60	10.85	Aurora Kinase A/B inhibitor	[33]
YM155 (Sepantronium Bromide)	12.25	10.78	Survivin inhibitor	[34]
Bleomycin sulfate	12.39	14.99	Glycopeptide antibiotic	[35]

## Data Availability

The data presented in this study are available in this article and the Supplementary Material.

## Supplementary Materials

The following supporting information can be downloaded at: https://www.mdpi.com/article/10.3390/cancers16234075/s1: Figure S1: Tool validation; Figure S2: Sample Tf v log[drug] curves used for AUC calculations; Table S1: AUC and SD values for all 448 compounds in Anti-Cancer compound library.
