# Supplementary material for: A High-Throughput Immune-Oncology Screen Identifies Immunostimulatory Properties of Cytotoxic Chemotherapy Agents in TNBC
Source: Cancers (Basel). 2024 Dec 5;16(23):4075. doi: 10.3390/cancers16234075 (PMC11639798; doi:10.3390/cancers16234075)
Supplement: Supplementary file 1 [file cancers-16-04075-s001.zip › cancers-3316542-supplementary.pdf]

Supplementary material

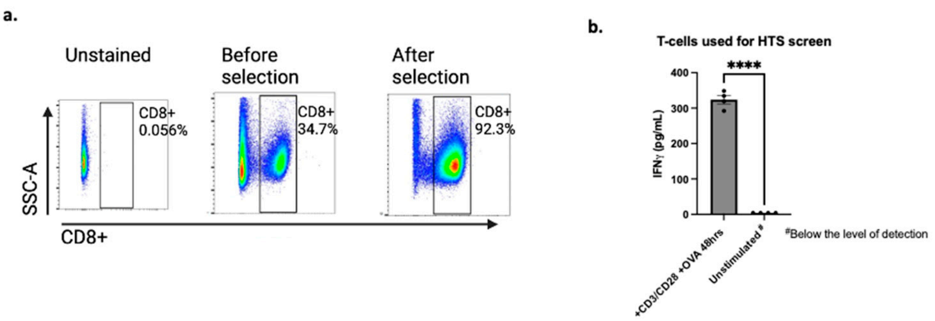

Figure S1 Tool validation **a.** Confirmation of enrichment of CD8+ T-cell population after magnetic bead selection. **b.** Confirmation of action status of CD8+ T-cells used in HTS assay. IFNy measured by ELISA analysis of cell culture supernatants. The unpaired student's t-test used for statistical comparison ****=p<0.0001.

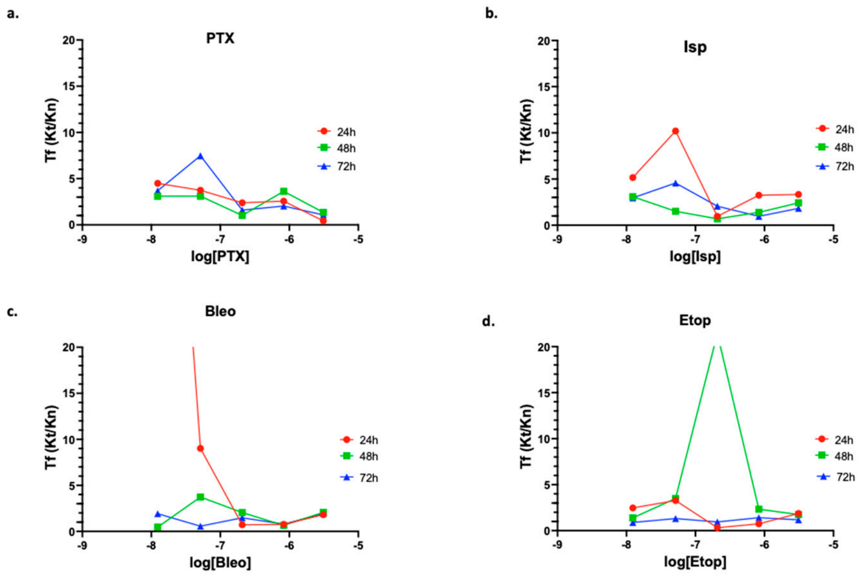

Figure S2 Sample Tf v log[drug] curves used for AUC calculations (a) PTX (b) Isp (c) Bleo (d) Etop

Table S1 AUC and SD values for all 448 compounds in Anti-Cancer compound library.

Drug name	24 hr AUC	48 hr AUC	72hr AUC	avg AUC	St Dev
MK-2866 (GTx-024)	11.65	5.82	4.29	7.25	3.89
BIIB021	4.06	5.92	3.92	4.63	1.12
AZ628	3.47	4.21	4.39	4.02	0.49
Altretamine (Hexalen)	35.50	5.33	2.93	14.59	18.15
Crizotinib (PF-02341066)	6.79	8.52	7.96	7.76	0.88
Lenalidomide	2.22	5.43	5.77	4.48	1.96
BIBR1532	5.33	8.41	8.74	7.49	1.88
Cediranib (AZD2171)	3.82	3.15	2.57	3.18	0.62
Cladribine	3.80	4.62	8.70	5.71	2.63
Bicalutamide (Casodex)	3.27	4.16	4.59	4.01	0.68
Sirtinol	2.50	3.12	2.20	2.61	0.47
Streptozotocin (Zanosar)	2.57	3.49	2.36	2.81	0.60
Cediranib (AZD2171)	4.01	4.59	6.67	5.09	1.40
Temsirolimus (Torisel)	5.40	3.50	4.19	4.36	0.96
ENMD-2076	22.12	3.23	2.09	9.15	11.25
Motesanib Diphosphate (AMG-706)	3.11	3.23	2.11	2.82	0.61
Nelarabine (Arranon)	4.10	7.41	5.68	5.73	1.66
Decitabine	4.95	3.67	3.79	4.14	0.71
Vinorelbine Tartrate	2.29	3.55	1.68	2.51	0.95
Zileuton	5.36	6.63	8.44	6.81	1.55
GSK1904529A	1.48	12.65	4.53	6.22	5.77
AUY922 (NVP-AUY922)	2.09	4.79	4.82	3.90	1.56
NPI-2358 (Plinabulin)	2.16	1.60	1.50	1.76	0.36
Trichostatin A	3.43	2.34	1.37	2.38	1.03
Ku-0063794	3.10	4.86	4.50	4.15	0.93
Ruxolitinib (INCB018424)	1.59	1.91	4.98	2.83	1.87
VS-5584 (SB2343)	2.86	3.86	1.72	2.82	1.07
AEE788 (NVP-AEE788)	30.06	3.95	2.40	12.14	15.54
Danusertib (PHA-739358)	4.23	7.59	3.87	5.23	2.05
JNJ 26854165 (Serdemetan)	2.97	3.88	3.46	3.44	0.45
1-(T-Butyl)-3-(2-((4-(diethylamino)butyl)amino)	2.88	2.99	1.90	2.59	0.60
PHA-665752	2.69	2.92	1.69	2.44	0.65
Vincristine Sulfate	3.30	2.24	4.76	3.43	1.26
XL-184 (Cabozantinib)	3.75	4.80	4.60	4.38	0.56
HSP990 (NVP-HSP990)	4.13	2.88	1.83	2.94	1.15
Aprepitant (MK-0869)	4.21	6.50	2.38	4.36	2.06
SRT1720	1.28	2.42	2.83	2.18	0.80
ABT-751 (E7010)	1.83	3.98	5.44	3.75	1.82
LY2228820	4.68	11.85	4.29	6.94	4.26
Belinostat (PXD101)	2.63	10.00	3.28	5.30	4.08

Mercaptopurine	2.27	3.88	3.88	3.34	0.93
CH5138303	2.23	2.65	3.19	2.69	0.48
PCI-32765 (Ibrutinib)	1.24	3.80	2.72	2.59	1.29
VU0415267-2	2.55	5.38	2.14	3.36	1.76
Barasertib (AZD1152-HQPA), AZD1152	2.48	2.21	3.19	2.63	0.50
Ispinesib (SB-715992)	11.30	3.82	6.05	7.06	3.84
Nedaplatin	3.82	5.01	2.22	3.68	1.40
JNJ-26481585	4.79	4.27	2.14	3.73	1.40
Pelitinib (EKB-569)	3.77	4.66	5.92	4.78	1.08
Flavopiridol (Alvocidib) HCl	3.54	6.10	3.28	4.31	1.56
Amuvatinib (MP-470)	1.74	3.40	2.72	2.62	0.84
PHA-793887	3.14	4.05	3.37	3.52	0.47
Bosutinib (SKI-606)	2.50	3.13	3.41	3.01	0.47
SB 525334	1.70	3.86	4.06	3.21	1.31
Docetaxel (Taxotere)	2.13	6.01	3.02	3.72	2.03
GSK690693	1.77	2.43	1.32	1.84	0.56
MK-1775	5.07	3.68	6.68	5.14	1.50
INK 128 (MLN0128)	1.95	3.26	6.02	3.74	2.08
AC480 (BMS-599626)	2.84	1.82	6.04	3.57	2.20
olaparib	2.94	1.70	5.00	3.21	1.66
Paclitaxel (Taxol)	6.76	6.01	8.20	6.99	1.11
Epothilone B (EPO906, Patupilone)	1.59	3.79	4.48	3.29	1.51
AT9283	3.01	11.49	7.70	7.40	4.25
SNS-314 Mesylate	1.36	2.63	6.04	3.35	2.42
PIK-93	3.74	4.35	5.34	4.48	0.81
Pazopanib	2.34	1.57	3.28	2.40	0.86
BI 2536	7.86	1.51	2.94	4.10	3.33
A-769662	80.24	2.26	8.53	30.34	43.33
Axitinib	4.32	4.80	8.99	6.03	2.57
Zibotentan (ZD4054)	2.56	3.36	4.96	3.62	1.22
CI-1040 (PD184352)	2.16	2.91	6.78	3.95	2.48
Xanthohumol	66.92	2.31	4.26	24.50	36.75
CHIR-99021 (CT99021) HCl	4.71	4.52	9.25	6.16	2.68
RO5126766 (CH5126766)	2.67	2.66	5.51	3.61	1.64
YM155 (Sepantronium Bromide)	5.55	24.69	6.51	12.25	10.78
Dexamethasone acetate	1.29	1.32	9.28	3.96	4.61
YM201636	9.77	6.11	7.65	7.85	1.84
TAPI-1	3.24	4.06	8.90	5.40	3.06
PD0325901	3.31	2.54	4.02	3.29	0.74
ESI-09	2.93	2.16	7.33	4.14	2.79
Chlorambucil	5.67	6.54	8.11	6.77	1.23
PharmaGSID_48511	3.00	2.39	5.69	3.69	1.75
ABT-888 (Veliparib)	3.79	2.37	6.06	4.07	1.86
Pomalidomide	5.91	3.60	5.65	5.05	1.27

Flavopiridol (Alvocidib) HCl	4.27	5.00	5.58	4.95	0.66
INH6	4.39	8.31	12.01	8.23	3.81
SB 216763	450.35	3.56	7.04	153.65	256.96
Santacruzamate A (CAY10683)	1.92	4.53	9.14	5.20	3.66
HS-173	4.69	4.67	4.79	4.72	0.06
Dacomitinib (PF299804, PF-00299804)	2.85	2.63	7.46	4.31	2.73
VU0485685-2	3.21	2.07	6.16	3.81	2.11
Tretinoin (Aberela)	2.08	11.16	8.74	7.33	4.70
Tipifarnib (Zarnestra)	3.75	3.45	5.36	4.19	1.03
EW-7197	3.30	2.75	7.26	4.44	2.46
Refametinib (RDEA119, Bay 86-9766)	2.10	2.44	4.71	3.08	1.42
Epacadostat (INCB024360)	2.25	3.22	5.38	3.62	1.61
PH-797804	8.36	4.68	6.56	6.53	1.84
ABT-737	1.14	1.43	4.58	2.38	1.91
MLN8237 (Alisertib)	2.60	1.51	5.71	3.27	2.18
Estradiol	4.17	1.73	6.72	4.21	2.49
3,5,7,8-Tetrahydro-2-	7.14	4.18	10.07	7.13	2.94
ZSTK474	3.44	3.14	6.13	4.24	1.65
Triciribine	5.08	33.54	9.20	15.94	15.38
NSC348884	3.36	10.40	9.61	7.79	3.86
3-Methyladenine (3-MA)	3.11	2.48	5.11	3.57	1.37
Linifanib (ABT-869)	2.04	2.45	3.54	2.68	0.78
Dovitinib (TKI-258, CHIR-258)	45.98	3.98	5.59	18.52	23.80
Ranolazine (Ranexa)	11.78	2.51	4.99	6.42	4.80
OSI-930	14.67	6.99	11.31	10.99	3.85
JNJ-38877605	3.47	2.93	5.36	3.92	1.28
Andarine (GTX-007)	2.42	3.12	5.15	3.56	1.42
HJC0350	2.42	2.01	4.35	2.92	1.25
Irinotecan HCl Trihydrate (Campto)	2.10	1.72	1.66	1.83	0.24
Nutlin-3	3.29	2.25	5.67	3.74	1.75
Cyclophosphamide monohydrate	1.58	2.88	2.59	2.35	0.68
Mitoxantrone Hydrochloride	2.38	2.70	2.47	2.52	0.16
PTC-209 HBr	0.99	1.76	1.88	1.54	0.48
Everolimus (RAD001)	3.13	3.93	2.68	3.25	0.64
PI-1840	2.36	1.79	1.74	1.96	0.34
VU0482726-2	1.45	3.04	1.99	2.16	0.81
Pioglitazone hydrochloride (Actos)	26.04	3.66	2.45	10.71	13.28
VU0823849-2	2.03	3.60	2.40	2.68	0.82
GSK461364	2.42	10.35	2.89	5.22	4.45
Bexarotene	1.93	1.43	1.51	1.62	0.26
BV-6	4.79	3.84	3.13	3.92	0.84
Capecitabine (Xeloda)	2.38	2.16	3.84	2.79	0.91
G-749	2.33	13.68	2.21	6.07	6.59
GDC-0623	8.50	2.75	10.67	7.31	4.10

R406(free base)	3.78	4.42	4.71	4.30	0.47
AZD6244 (Selumetinib)	2.37	2.58	3.05	2.67	0.35
Momelotinib (CYT387)	2.04	2.50	1.68	2.07	0.41
VU0814307-2	1.48	1.42	1.60	1.50	0.09
VU0656797-2	2.46	2.57	2.19	2.41	0.19
Nintedanib (BIBF 1120)	2.05	2.55	4.72	3.11	1.42
PX-478 2HCl	2.53	6.25	1.64	3.47	2.44
Tasquinimod	1.59	1.40	2.04	1.68	0.33
Ezetimibe (Zetia)	3.19	1.50	2.16	2.28	0.85
PI-103	2.25	2.88	3.25	2.79	0.51
Mifepristone (Mifeprex)	1.08	1.96	2.16	1.73	0.58
TW-37	2.75	2.58	1.84	2.39	0.48
Licochalcone A	1.17	2.53	2.68	2.13	0.83
Gefitinib (Iressa)	3.42	13.37	2.19	6.33	6.13
AZD6738	5.83	1.98	2.22	3.34	2.16
Fulvestrant (Faslodex)	2.14	2.05	2.73	2.31	0.37
Roscovitine	2.52	2.16	2.00	2.22	0.27
SB-203580	1.10	1.83	2.49	1.81	0.70
BIRB 796 (Doramapimod)	2.74	2.10	5.03	3.29	1.54
Ganetespib (STA-9090)	1.23	1.90	1.60	1.58	0.34
Masitinib (AB1010)	79.46	1.13	2.04	27.54	44.96
Rapamycin (Sirolimus)	1.34	2.38	1.71	1.81	0.53
tretazicar	1.45	1.44	1.74	1.54	0.17
Clofarabine	1.87	2.26	1.43	1.85	0.41
Saracatinib (AZD0530)	2.65	1.91	1.83	2.13	0.45
17-AAG (Tanespimycin)	1.09	1.70	5.31	2.70	2.28
Estrone	12.64	2.09	1.31	5.35	6.33
AZD7762	2.14	1.43	4.90	2.82	1.83
PCI-24781, CRA-02478	2.08	1.75	1.92	1.92	0.17
GDC-0941	1.63	1.97	1.82	1.81	0.17
cucurbitacin B	1.05	1.34	1.28	1.22	0.15
Fludarabine Phosphate (Fludara)	2.20	1.55	1.64	1.80	0.35
VX680	2.21	2.70	3.38	2.77	0.59
Elesclomol (STA-4783)	1.41	2.97	2.39	2.26	0.79
Azathioprine (Azasan, Imuran)	1.96	1.64	1.68	1.76	0.17
PF 573228	1.80	2.36	1.71	1.96	0.35
VU0549317-2	1.85	1.35	1.65	1.61	0.25
MK-2206 dihydrochloride	1.72	1.91	1.27	1.63	0.33
FH535	2.29	3.73	3.07	3.03	0.72
JNJ-7706621	2.37	1.60	7.65	3.87	3.29
Ixazomib citrate	5.78	1.69	1.79	3.09	2.33
EX 527, SEN0014196	4.66	4.36	2.94	3.98	0.92
Disulfiram (Antabuse)	1.78	2.40	2.28	2.15	0.33
Tie2 kinase inhibitor	6.69	2.98	2.13	3.94	2.43

Carmofur	13.05	2.07	2.13	5.75	6.32
Afuresertib (GSK2110183)	4.00	5.39	2.57	3.99	1.41
PRI-724	2.93	2.48	6.36	3.92	2.12
Topotecan HCl	4.86	3.99	3.13	4.00	0.86
Degrasyn (WP1130)	1.79	3.13	2.43	2.45	0.67
Aminoglutethimide (Cytadren)	3.18	3.90	3.48	3.52	0.36
Toremifene Citrate (Fareston, Acapodene)	1.81	2.58	1.80	2.06	0.45
Sonidegib	5.19	2.81	2.37	3.46	1.52
Dexamethasone	230.56	1.78	3.45	78.60	131.61
4SC-202	3.44	3.06	3.37	3.29	0.20
ML323	1.46	1.94	6.47	3.29	2.76
Celecoxib	2.17	1.63	1.90	1.90	0.27
Phloretin	9.66	1.62	15.89	9.05	7.15
Mesna	3.80	2.73	2.61	3.04	0.66
RO4929097	1.77	1.83	3.28	2.29	0.85
idelalisib	2.75	9.24	3.87	5.29	3.47
Ponatinib (AP24534)	6.09	5.83	3.44	5.12	1.46
LY2584702 tosylate	7.71	3.96	3.81	5.16	2.21
Rigosertib (ON-01910)	12.23	5.95	2.37	6.85	4.99
Floxuridine	3.48	2.54	2.59	2.87	0.53
KU-60019	3.37	2.77	2.67	2.94	0.38
Hydroxyurea (Cytodrox)	4.58	6.75	2.37	4.57	2.19
SGI-1776 free base	3.54	2.60	2.16	2.77	0.70
Cyclosporin A (Cyclosporine A)	13.45	2.00	2.20	5.88	6.55
Regorafenib (BAY 73-4506)	5.78	3.10	1.84	3.57	2.01
BI-847325	2.56	2.57	2.67	2.60	0.06
Fludarabine (Fludara)	4.28	3.55	2.59	3.48	0.85
Mycophenolate mofetil (CellCept)	10.76	2.36	4.35	5.83	4.39
BKM120 (NVP-BKM120)	3.73	3.07	3.65	3.49	0.36
Lapatinib	3.34	3.11	2.49	2.98	0.44
EPIGALLOCATECHIN-3-MONOGALLATE	10.09	4.19	4.88	6.38	3.23
LY2603618 (IC-83)	2.26	3.63	2.96	2.95	0.69
Raltitrexed (Tomudex)	4.06	5.75	3.49	4.43	1.17
LTX-315	2.65	1.89	2.15	2.23	0.38
Divalproex sodium	14.48	8.35	2.34	8.39	6.07
CYC116	3.21	23.86	7.74	11.60	10.85
tanshinone	2.57	2.94	3.79	3.10	0.63
TAME	3.26	4.87	2.49	3.54	1.22
chrysophanic acid	4.43	2.63	2.91	3.32	0.97
Mitoxantrone Hydrochloride	1.21	1.59	8.55	3.78	4.13
Tivozanib (AV-951)	4.12	3.39	2.11	3.21	1.02
XMD8-92	1.95	2.98	4.86	3.26	1.47
Thalidomide	3.19	2.94	2.39	2.84	0.41
VU0656495-2	3.54	2.19	1.88	2.54	0.88

Lonidamine	2.69	4.14	2.64	3.16	0.85
Febuxostat (Uloric)	2.30	2.59	2.29	2.40	0.17
TAK-733	2.33	3.00	2.80	2.71	0.35
Prednisone (Adasone)	2.66	2.78	1.43	2.29	0.75
ML264	1.60	1.88	2.66	2.05	0.55
MI-773 (SAR405838)	1.84	3.15	1.97	2.32	0.72
Doxorubicin (Adriamycin)	1.90	3.17	2.22	2.43	0.66
C-DIM12	2.84	2.99	2.08	2.64	0.49
Betapar (Meprednisone)	3.26	2.39	2.57	2.74	0.46
Busulfan (Myleran, Busulfex)	3.05	2.34	2.34	2.58	0.41
Allopurinol	2.35	2.94	2.23	2.51	0.38
BMS 777607	2.43	12.43	3.16	6.01	5.57
Palbociclib	1.36	3.19	2.23	2.26	0.92
CH5183284 (Debio-1347)	1.94	2.33	2.12	2.13	0.20
Exemestane	4.65	3.61	3.13	3.79	0.78
YH239-EE	1.18	1.56	1.25	1.33	0.20
Azacitidine (Vidaza)	3.28	2.69	2.10	2.69	0.59
Gossypol	2.60	2.53	3.01	2.71	0.26
Ixazomib (MLN2238)	4.17	2.63	7.68	4.83	2.59
Hydrocortisone (Cortisol)	2.81	1.93	2.76	2.50	0.49
miltefosine	8.14	3.93	12.02	8.03	4.05
Dacarbazine (DTIC-Dome)	3.03	2.32	1.49	2.28	0.77
Methotrexate	4.80	3.19	2.46	3.48	1.20
Pilaralisib (XL147)	1.12	1.91	1.58	1.54	0.39
Fluvastatin sodium (Lescol)	5.50	4.00	2.76	4.09	1.38
GW3965 HCl	4.50	2.66	1.93	3.03	1.33
BI6727 (Volasertib)	3.79	1.73	4.88	3.47	1.60
Simvastatin (Zocor)	2.25	2.12	2.26	2.21	0.08
APTSTAT3-9R	2.85	2.45	2.44	2.58	0.23
2-Methoxyestradiol	2.15	3.13	1.96	2.41	0.62
Oxaliplatin (Eloxatin)	0.90	2.19	1.79	1.63	0.66
Nutlin-3A	1.74	1.77	2.37	1.96	0.36
AZD1480	22.28	2.97	1.95	9.07	11.46
AZD8055	1.93	2.64	2.57	2.38	0.39
Oridonin (Isodonol)	2.07	6.32	1.83	3.41	2.52
Maraviroc	2.60	6.64	1.57	3.60	2.68
AT7519	2.48	14.44	1.70	6.20	7.14
Ftorafur	3.46	3.71	2.52	3.23	0.63
Letrozole	2.38	2.03	2.21	2.21	0.18
WIKI4	1.84	2.48	1.13	1.82	0.68
OSI-420 (Desmethyl Erlotinib)	2.50	1.81	1.78	2.03	0.41
1-(4-dimethylaminomethylphenyl)	5.07	3.01	2.80	3.63	1.25
Telatinib (BAY 57-9352)	3.94	2.66	1.92	2.84	1.02
DAPT (GSI-IX)	2.37	5.94	2.48	3.60	2.03

Mitomycin	2.44	3.69	2.58	2.90	0.68
Bafetinib (INNO-406)	3.51	3.34	3.13	3.33	0.19
HO-3867	2.40	3.14	2.19	2.58	0.50
ODM-201	1.92	1.15	2.44	1.83	0.65
Mycophenolic (Mycophenolate)	2.87	2.88	2.00	2.58	0.50
Formestane	4.32	3.28	2.74	3.44	0.80
Rosiglitazone HCl	3.73	2.27	2.08	2.70	0.90
Medroxyprogesterone acetate	2.56	2.72	1.91	2.40	0.43
VER-50589	2.39	3.56	1.77	2.57	0.91
Doxercalciferol (Hectorol)	2.12	1.89	2.54	2.18	0.33
TIC10	34.48	1.12	3.22	12.94	18.68
Dovitinib (TKI-258, CHIR-258)	1.60	2.58	2.73	2.31	0.61
Triamcinolone Acetonide	3.40	2.16	2.30	2.62	0.68
limonin	9.67	2.77	1.86	4.77	4.27
DCC-2036 (Rebastinib)	3.81	4.11	1.58	3.17	1.38
Omipalisib (GSK2126458, GSK458)	3.49	3.88	1.25	2.87	1.42
PF-06463922	2.33	1.74	1.68	1.92	0.36
SB939 (Pracinostat)	1.58	1.88	1.98	1.81	0.21
PU-H71	1.62	1.42	1.96	1.67	0.27
SGI-7079	3.12	3.08	2.20	2.80	0.52
GW 4064	3.65	3.56	2.60	3.27	0.58
GSK923295	4.36	3.71	1.92	3.33	1.26
KU-55933 (ATM Kinase Inhibitor)	14.69	3.25	2.44	6.79	6.86
Fingolimod	6.97	2.82	2.63	4.14	2.45
Ruxolitinib (INCB018424)	3.84	1.34	1.68	2.29	1.36
Methazolastone	5.28	4.15	2.50	3.97	1.40
CNX-2006	2.39	18.12	2.29	7.60	9.11
Bleomycin sulfate	29.68	4.65	2.85	12.39	15.00
TPCA-1	3.79	3.52	4.26	3.86	0.37
SH-4-54	3.98	2.01	2.17	2.72	1.09
LY 294002	2.76	2.53	2.76	2.68	0.13
PF-3758309	2.68	1.97	6.34	3.66	2.35
XL765	2.14	2.67	2.34	2.38	0.27
Vemurafenib (PLX4032)	4.46	2.24	2.47	3.06	1.22
Osimertinib	2.94	3.39	2.63	2.99	0.38
CPI-203	4.17	4.26	3.27	3.90	0.55
Vitamin E	2.70	2.38	1.77	2.28	0.47
trametinib	2.96	2.91	1.84	2.57	0.63
Mocetinostat (MGCD0103)	4.09	2.75	12.02	6.28	5.01
Daunorubicin HCl (Daunomycin HCl)	2.69	2.80	2.56	2.68	0.12
WZ4002	4.76	3.38	3.04	3.72	0.91
Megestrol Acetate	6.03	3.28	2.83	4.05	1.73
KX2-391	4.24	4.27	3.36	3.96	0.52
AT7519	3.19	2.16	1.86	2.40	0.70

CW069	7.26	2.97	3.47	4.57	2.35
Sorafenib (Nexavar)	2.26	2.55	3.04	2.61	0.39
SNS-032 (BMS-387032)	4.65	3.66	1.76	3.36	1.47
Brivanib (BMS-540215)	2.48	1.67	2.94	2.36	0.65
Anastrozole	5.65	3.46	3.26	4.13	1.33
AT13148	1.97	3.24	2.16	2.46	0.69
Fedratinib (SAR302503, TG101348)	3.99	4.36	2.73	3.69	0.86
Enzastaurin (LY317615)	4.10	4.18	3.19	3.82	0.55
PF-3845	5.67	4.95	2.41	4.34	1.71
Entinostat	5.11	1.67	2.47	3.09	1.80
BMS-536924	5.58	3.76	2.81	4.05	1.41
AZ5104	3.99	3.28	8.43	5.23	2.79
Irinotecan HCl Trihydrate (Campto)	4.07	2.42	2.48	2.99	0.94
CH5132799	5.73	3.25	3.69	4.22	1.32
PF-562271	3.95	2.57	2.30	2.94	0.88
CB-839	2.78	2.62	3.10	2.83	0.24
Tofacitinib citrate (CP-690550 citrate)	5.73	3.13	4.35	4.41	1.30
4-(4-(Benzo[d][1,3]dioxol-5-yl)	6.20	3.22	10.52	6.65	3.67
Lapatinib	6.97	3.28	2.72	4.33	2.31
WZB117	5.92	5.90	2.68	4.83	1.86
Bendamustine HCL	6.27	4.69	3.35	4.77	1.46
Anagrelide HCl	5.52	6.03	4.10	5.22	1.00
Triptolide	9.59	2.98	1.74	4.77	4.22
Flutamide (Eulexin)	3.38	3.91	2.18	3.16	0.89
JNK-IN-8	6.11	4.16	2.70	4.32	1.71
SU11274	3.38	2.99	2.14	2.84	0.63
Sotrastaurin (AEB071)	4.41	3.24	1.96	3.20	1.22
AZD1208	6.50	3.58	2.91	4.33	1.91
Etoposide (VP-16)	3.95	17.35	2.83	8.04	8.08
GSK2334470	5.20	3.78	2.61	3.86	1.30
VU0656030-2	2.83	4.44	1.73	3.00	1.36
Torin 2	3.16	3.11	2.22	2.83	0.53
IOX1	3.87	2.39	1.65	2.64	1.13
ABC294640	51.87	2.84	2.50	19.07	28.41
LY2874455	4.17	2.24	1.76	2.72	1.28
TMP269	1.94	2.83	2.44	2.40	0.45
Quizartinib (AC220)	1.96	2.68	1.89	2.18	0.44
Iniparib (BSI-201)	3.14	4.77	1.47	3.13	1.65
OSI-906 (Linsitinib)	3.60	2.67	2.34	2.87	0.65
NVP-BSK805 2HCl	1.74	1.80	1.72	1.75	0.04
SF1670 (PTEN inhibitor)	6.75	2.86	2.82	4.14	2.26
6H05	2.48	5.17	2.03	3.22	1.70
BAY 11-7082	2.67	0.99	1.83	1.83	0.84
GDC-0879	2.12	2.82	3.53	2.82	0.70

danthron	4.89	4.32	5.69	4.97	0.69
Crenolanib (CP-868596)	2.75	2.67	2.05	2.49	0.38
GSK650394	2.40	3.13	1.91	2.48	0.61
MDV3100 (Enzalutamide)	4.44	3.54	2.42	3.46	1.01
Gemcitabine (Gemzar)	4.39	4.10	2.18	3.56	1.20
Sunitinib Malate (Sutent)	2.35	2.21	2.84	2.47	0.33
BTZ043 racemate	3.21	3.33	2.36	2.97	0.53
Lenvatinib	23.75	2.93	2.27	9.65	12.22
Evista (Raloxifene Hydrochloride)	2.49	2.59	2.77	2.62	0.14
ABT-263 (Navitoclax)	3.40	2.71	2.09	2.73	0.66
17-DMAG HCl (Alvespimycin)	2.64	1.63	2.04	2.11	0.51
Quercetin	4.70	2.94	2.65	3.43	1.11
Nilotinib (AMN-107)	1.98	1.38	1.56	1.64	0.31
Dasatinib (BMS-354825)	3.32	2.89	1.87	2.69	0.74
Y-27632 2HCl	24.86	6.25	1.89	11.00	12.20
LDC000067	4.01	2.93	2.22	3.05	0.90
PF-04217903	1.77	2.85	1.71	2.11	0.64
Vorinostat (SAHA)	2.93	2.60	2.08	2.54	0.43
Lomustine (CeeNU)	3.82	4.67	1.90	3.47	1.42
Nutlin-3A	1.38	2.15	1.71	1.75	0.38
CEP33779	3.74	2.57	1.60	2.64	1.07
PD173955	5.13	3.70	2.83	3.89	1.16
Pexmetinib (ARRY-614)	3.84	3.38	2.12	3.12	0.89
PF-543	7.42	2.64	1.86	3.98	3.01
RSL3	3.16	2.46	1.90	2.51	0.63
Endoxifen HCl	4.22	3.29	1.81	3.11	1.21
4-Hydroxytamoxifen	1.29	2.83	2.27	2.13	0.78
sulfabenzamide	4.00	3.58	2.92	3.50	0.54
AZD8186	4.09	2.69	1.85	2.88	1.13
PND-1186 (VS-4718)	2.28	1.49	2.73	2.17	0.63
DalcetrapibèJTT-705é	4.88	4.43	3.50	4.27	0.70
Phosphoramidon Disodium Salt	3.90	2.43	1.71	2.68	1.11
Teniposide (Vumon)	2.72	6.58	2.54	3.94	2.28
Afatinib (BIBW2992)	2.90	2.72	1.91	2.51	0.52
Z-VAD-FMK	87.20	12.35	6.33	35.29	45.05
VU0656122-2	8.56	3.51	2.83	4.97	3.13
CX-6258 HCl	3.59	4.14	2.02	3.25	1.10
ARQ 621	2.96	2.73	1.13	2.27	0.99
Deltarasin	4.79	3.62	2.33	3.58	1.23
Geldanamycin	2.62	2.55	2.14	2.44	0.26
PFK15	3.42	2.75	1.66	2.61	0.89
(E)-N'-(3-Allyl-2-hydroxybenzylidene)	3.21	1.60	1.86	2.23	0.86
DMXAA (Vadimezan)	2.87	6.66	4.01	4.51	1.94
MI-2 (MALT1 inhibitor)	4.77	2.83	2.45	3.35	1.25

CYT997	3.53	1.64	1.55	2.24	1.12
Adrucil (Fluorouracil)	1.77	6.94	1.97	3.56	2.93
INCB024360 analogue	4.77	1.60	1.73	2.70	1.79
Apitolisib (GDC-0980, RG7422)	3.60	3.13	2.43	3.05	0.59
CUDC-101	7.14	4.77	7.03	6.31	1.34
Tamoxifen Citrate (Nolvadex)	1.25	1.29	1.41	1.32	0.08
cx-4945 (Silmitasertib)	3.38	3.23	6.21	4.27	1.68
Alpelisib (BYL719)	4.31	2.73	2.94	3.33	0.86
Epothilone A	4.53	2.60	6.68	4.60	2.04
Calcium levofolinate (Calcium Folinate)	4.51	4.31	3.12	3.98	0.75
Duvelisib (IPI-145, INK1197)	3.33	2.53	1.94	2.60	0.70
PI3K/HDAC Inhibitor I	5.20	2.86	2.08	3.38	1.62
BMS 794833	3.24	3.06	1.88	2.73	0.74
Fosbretabulin (Combretastatin A4 Phosphate,	3.39	3.93	3.32	3.54	0.33
CZC24832	7.81	3.79	7.83	6.48	2.33
(5E)-5-[(2,2-Difluoro-1,3-benzodioxol-5-YL)	4.86	3.24	2.79	3.63	1.09
INH1	6.12	1.56	1.73	3.14	2.59
Turofexorate Isopropyl (XL335)	4.61	3.16	2.39	3.39	1.13
TG100-115	3.62	3.71	1.34	2.89	1.34
3-Methyladenine (3-MA)	3.91	6.32	2.95	4.39	1.74
GF 109203X	2.81	1.87	1.77	2.15	0.57
SB-3CT	5.89	2.12	1.87	3.29	2.25
GDC-0349	3.70	2.42	2.37	2.83	0.76
PIK-294	3.94	2.18	1.94	2.68	1.09
GSK2606414	16.55	1.56	1.12	6.41	8.79
RG108	4.13	3.12	2.86	3.37	0.67
VPS34-IN1	2.44	1.32	1.55	1.77	0.59
AMG319	4.10	2.54	1.77	2.80	1.19
K-Ras(G12C) inhibitor 9	3.23	2.19	2.85	2.75	0.53
YO-01027 (Dibenzazepine)	3.87	3.37	2.50	3.25	0.70
WAY-600	4.13	3.42	2.39	3.31	0.88
AS-252424	4.82	13.78	2.18	6.93	6.08
Clioquinol	3.37	2.19	1.92	2.49	0.77
Nocodazole	6.13	2.90	2.86	3.96	1.88
AZD2014	2.74	2.70	1.79	2.41	0.54
MLN2480	11.83	2.80	2.37	5.66	5.34
MK-0752	20.33	4.37	2.49	9.06	9.80
AMG 900	4.57	2.40	1.84	2.94	1.44
AZD6482	5.31	4.31	3.79	4.47	0.77
LY3023414	4.63	4.92	2.04	3.86	1.58
RG7112 (RO5045337)	3.40	3.00	2.03	2.81	0.70
AZ 3146	2.60	2.42	2.10	2.37	0.25
Asiatic acid	4.19	2.48	1.36	2.68	1.43
Palomid 529	4.00	4.28	3.94	4.08	0.18

VU0656492-2	5.07	3.38	2.12	3.52	1.48
Isotretinoin	19.19	4.02	2.95	8.72	9.08
Chloroquine	3.44	2.54	2.46	2.81	0.55
PD-98059	7.00	2.09	2.01	3.70	2.86
Galunisertib (LY2157299)	4.11	2.22	2.11	2.81	1.13
Ifosfamide	4.45	3.25	2.04	3.25	1.20
A66	4.23	2.05	2.37	2.88	1.18
NVP-BGT226	4.64	4.61	2.33	3.86	1.33
WYE-354	2.99	2.00	1.33	2.11	0.84
Imatinib (Gleevec)	3.23	2.57	1.91	2.57	0.66
CCT128930	3.50	2.56	1.54	2.53	0.98
GSK2292767	4.54	4.41	2.94	3.96	0.89
